# Cinacalcet inhibition of neuronal action potentials preferentially targets the fast inactivated state of voltage-gated sodium channels

**DOI:** 10.3389/fphys.2022.1066467

**Published:** 2022-12-19

**Authors:** Jamie S. Lindner, Salil R. Rajayer, Briana J. Martiszus, Stephen M. Smith

**Affiliations:** ^1^ Section of Pulmonary and Critical Care Medicine, VA Portland Health Care System, Portland, OR, United States; ^2^ Department of Medicine, Division of Pulmonary, Allergy and Critical Care Medicine, Oregon Health & Science University, Portland, OR, United States

**Keywords:** sodium channel, voltage-gated sodium channel, action potential, calcium-sensing receptor, CaSR, cinacalcet

## Abstract

Voltage-gated sodium channel (VGSC) activation is essential for action potential generation in the brain. Allosteric calcium-sensing receptor (CaSR) agonist, cinacalcet, strongly and ubiquitously inhibits VGSC currents in neocortical neurons *via* an unidentified, G-protein-dependent inhibitory molecule. Here, using whole-cell patch VGSC clamp methods, we investigated the voltage-dependence of cinacalcet-mediated inhibition of VGSCs and the channel state preference of cinacalcet. The rate of inhibition of VGSC currents was accelerated at more depolarized holding potentials. Cinacalcet shifted the voltage-dependence of both fast and slow inactivation of VGSC currents in the hyperpolarizing direction. Utilizing a simple model, the voltage-dependence of VGSC current inhibition may be explained if the affinity of the inhibitory molecule to the channel states follows the sequence: fast-inactivated > slow-inactivated > resting. The state dependence of VGSC current inhibition contributes to the non-linearity of action potential block by cinacalcet. This dynamic and abundant signaling pathway by which cinacalcet regulates VGSC currents provides an important voltage-dependent mechanism for modulating central neuronal excitability.

## Introduction

Voltage-gated sodium channels (VGSC) are essential for the action potential generation and propagation that is central to physiological function in excitable cells (Hodgkin and Huxley, 1952; Hille, 2001). The complex, membrane potential-dependent gating behavior places them at the center of rapid, dynamic intracellular signaling and emphasizes their role as key players in the function of neurons, skeletal muscle cells, and the vast majority of cardiac cells (Karoly et al., 2010). Even slight disturbances in the gating behavior of VGSCs may unbalance excitability, and give rise to various conditions such as epilepsy, cardiac arrhythmia, seizure, and chronic pain (Montini et al., 2018). VGSCs are targets for a wide range of important drugs used as local anesthetics, antiarrhythmics, and anticonvulsants that operate by reducing excitability in cardiac and central nervous tissue (Catterall, 1999; Hille, 2001; Nau and Wang, 2004).

Specificity of action of different VGSC inhibitors across cell types arises from a number of factors. Tissue-specific variation in VGSC subtypes is an important contributor to the selectivity of effect since VGSC modulator affinity may vary with VGSC subunit isoform (Mantegazza et al., 2005; England and de Groot, 2009; Okura et al., 2014). Direct inhibitors that act by pore blocking of VGSCs (Guo et al., 1987) or by preferential binding to specific VGSC states may also influence the tissue specific effectiveness of the agent. Normal VGSC operation involves the channel cycling through a number of distinct functional states including resting, activated, fast-inactivated, and slow-inactivated (Montini et al., 2018). VGSC inhibitors may have different affinities for each state, often with a preference for the open or inactivated state (Kuo and Bean, 1994; Karoly et al., 2010; Jo and Bean, 2011; Bagal et al., 2015; Jo and Bean, 2017; Sait et al., 2020). As the proportion of channels populating different states is controlled by cell membrane potential, this state-dependence of binding and inhibition confers voltage-dependence to the inhibition (Bagal et al., 2015). If the inhibitor binds preferentially to a particular channel state, it will be more effective in tissues with membrane potentials that increase the fraction of channels in that state (Kuo and Bean, 1994; Karoly et al., 2010; Theile et al., 2016). Indirect VGSC inhibitors, may operate *via* G-protein coupled receptors (GPCR), and thereby confer selectivity of effect (Cantrell et al., 1996; Mattheisen et al., 2018). In this case, regional specificity may arise from variable expression of the GPCR or the downstream signaling pathway, as either will influence whether the cells are modulated by the VGSC inhibitor. The degree of VGSC inhibition by G-protein regulation ranges from 10% to 100% depending on brain regions and GPCR identity (Cantrell et al., 1996; Cantrell et al., 1997; Carr et al., 2002; Carlier et al., 2006; Mattheisen et al., 2018).

Cinacalcet, a calcium-sensing receptor (CaSR) allosteric agonist is used to control hyperparathyroidism and hypercalcemia (Zitt et al., 2011; Chertow et al., 2012; Leere et al., 2017) in a number of clinical scenarios. Recently cinacalcet was identified as an indirect inhibitor of VGSC currents. The effect of cinacalcet was unaffected by CaSR deletion but was blocked by GDPβS indicating an indirect pathway mediated by G-proteins that was independent of CaSR (Mattheisen et al., 2018). Prolonged application of cinacalcet slowly but completely inhibited VGSC current amplitude in 100% of neocortical neurons studied indicating the underlying signaling pathway has high abundance and efficacy. Greater characterization of the molecular mechanisms underlying this strong, CaSR-independent pathway is crucial to provide insight into how it shapes neuronal excitability and determine its physiological role. Additionally, the unusual kinetics and mechanism of VGSC inhibtion by cinacalcet points to a signaling pathway that, if harnessed, might usefully expand the therapeutic armamentarium of sodium channel inhibitors.

To better understand how neuronal excitability is affected by the pathway utilized by cinacalcet to inhibit VGSC currents, we studied how VGSC properties are affected following the application of this drug to neocortical neurons. Here, we show that cinacalcet activity is enhanced at more depolarized holding potentials, indicating a preference of an unidentified downstream inhibitory molecule (X) for the inactivated state. Reversal of cinacalcet-mediated inhibition of VGSC currents *via* prolonged hyperpolarization indicated that the mechanism involves stabilization of the inactivated state(s) and slows recovery from these states. We investigated the correlation between the development of inactivation and the kinetics of inhibition by cinacalcet. The data support a model indicating that X binds to the various channel states with the preference fast-inactivated > slow-inactivated > resting state.

## Materials and methods

### Ethical approval

All animal procedures were approved by VA Portland Health Care System Institutional Animal Care and Use Committee (IRBNetID: 1635414–4) in accordance with the US Public Health Service policy on Humane Care and Use of Laboratory Animals and the National Institutes of Health Guide for the Care and Use of Laboratory Animals.

### Preparation of neuronal cultures

Neocortical neurons were isolated from 1 to 2 day old postnatal mouse pups of either sex as described previously (Martiszus et al., 2021; Ritzau-Jost et al., 2021). Animals were decapitated following general anesthesia with isoflurane and cerebral cortices were removed. Cortices were incubated in trypsin and DNase (5 mg/ml and 0.1 mg/ml for 5 min at 34°C) and dissociated with heat polished pipettes. Dissociated cells were maintained in MEM plus 5% FBS on glass coverslips in an incubator (humidified air and 5% CO_2_) at 37 °C. Cytosine arabinoside (4 µM) was added 48–72 h after plating to limit glial division. Cells were used, unless otherwise stated, after 7–35 days in culture.

### Electrophysiological recordings

Cells were visualized with an inverted microscope (Leica DM IRB or Olympus IX70). Whole-cell voltage-clamp recordings were made from cultured neocortical neurons using an Axopatch 200B amplifier with 60%–80% series resistance compensation. Current clamp recordings were made using a Heka EPC10 amplifier. The preparation was continously perfused with solution which contained (in mM) 150 NaCl, 4 KCl, 10 HEPES, 10 glucose, 1.1 MgCl_2_, 1.1 CaCl_2_, pH 7.35 with NaOH. Synaptic transmission was blocked by the addition of (in µM) 10 CNQX, 10 Gabazine, and 50 APV in extracellular bath solution. In current clamp recordings, 2 mM CsCl was added to the extracellular bath solution to reduce contributions of HCN. Voltage-clamp recordings were made using a caesium methanesulfonate intraceullar solution containing (mM) 135 caesium methansulfonate, 1.8 EGTA, 10 HEPES, 4 MgCl_2_, 0.2 CaCl_2_, 0.3 NaGTP, 4 NaATP, 14 phosphocreatine disodium, pH 7.2 with TEA hydroxide. Current-clamp recordings were made using potassium-gluconate containing intracellular solution containing (mM) 135 Potassium gluconate, 10 HEPES, 4 MgCl_2_, 0.3 NaGTP, 4 NaATP, 10 phosphocreatine disodium, pH 7.2 with potassium hydroxide. The electrode resistance in voltage- and current-clamp recordings were usually 2–3 MΩ and 6–8 MΩ respectively. Voltages have been corrected for liquid junction potentials. All experiments were performed at room temperature (21–23°C).

### Microarray analysis of gene expression in neocortical cultures

Neocortical cultures were prepared as above but plated in 25 cm^2^ flasks for gene profiling experiments. After 14 days in culture, cells were collected in RLT lysis buffer with 1% 2-mercaptoethanol in 2 ml RB (Qiagen) tubes and stored at −80°C. RNA isolation and microarray assays were performed in the OHSU Gene Profiling Shared Resource. RNA was extracted using the RNeasy Mini Kit (Qiagen) following the manufacturer’s recommended protocol. RNA quality was verified by Bioanalyzer assay (Agilent Technologies). Labeled target cDNA was prepared using the Applied Biosystems WT Plus protocol with an input of 100 ng total RNA. Processed samples were hybridized to a GeneChipTM Clariom S Mouse Array (Affymetrix/Applied Biosystems). Image processing was performed using Affymetrix Command Console (AGCC) v.3.1.1 software and expression analysis was performed using Affymetrix Expression Console software ver.1.4.1.46. The microarray data are available at NCBI GEO (Accession: PRJNA901951; GEO: GSE218028).

### Data acquisition and analysis

Whole cell voltage-clamp recordings were made using an Axopatch 200B Amplifier, filtered at 5 kHz using a Bessel filter, and sampled at 20 kHz during acquisition. Whole cell current-clamp recordings were made using a Heka EPC 10 amplifier, filtered at 2.9 kHz using a Bessel filter and sampled at 20 kHz during acqusition. Series resistance compensation was performed manually prior to acquisition. Analysis was performed using Igor Pro 8 (Wavemetrics, Lake Oswego, OR). Inactivation curves were generated by plotting the normalized peak VGSC current (I_Na_(norm.)) versus conditioning voltage (V) which was fit using the Boltzmann function:

$$I_{Na}\left(norm.\right)=I_{Res}+\left(1-{\;I}_{Res}\right)/\left(1+\exp\left(-z\frac{\left(V-V_{0.5}\right)}{24}\right)\right)$$

where I_Res_, z, and V_0.5_ represent the residual current resistant to inactivation, the apparent valence, and the mid-point of inactivation respectively. The maximum I_Na_(norm.) value predicted by the Boltzmann function was used for normalization. In current clamp experiments, input resistance was measured using the steady state voltage deflection elicited by a 70 pA current injection. Action potential properties were obtained by analyzing traces off-line with IgorPro macros. Action potential threshold was measured as the point at which dV/dt reached 20 mV/ms. Action potential amplitude was defined as the voltage difference between threshold and peak. Action potential half-width was defined as the interval between rising and falling phases of the spike at the point halfway between the peak and the holding potential. All data values were reported as mean (± SEM) or median, if not normally distributed. Statistical significance was determined with appropriate parametric or non-parametric tests (GraphPad Prism 8) as described in the figure legends.

### Solution application

Solutions were applied by gravity from a glass capillary (1.2 mm outer diameter) placed 1–2 mm from the neuron under study. Solutions were switched manually using a low dead volume manifold upstream of the glass capillary. CNQX and Gabazine were supplied by Abcam. Creatine Phosphate was supplied by Santa Cruz Biotech. Cinacalcet was supplied by Toronto Research Chemicals. All other reagents were supplied by Sigma-Aldrich.

## Results

### Voltage-dependence of cinacalcet-induced inhibition of VGSCs

**FIGURE 1:**
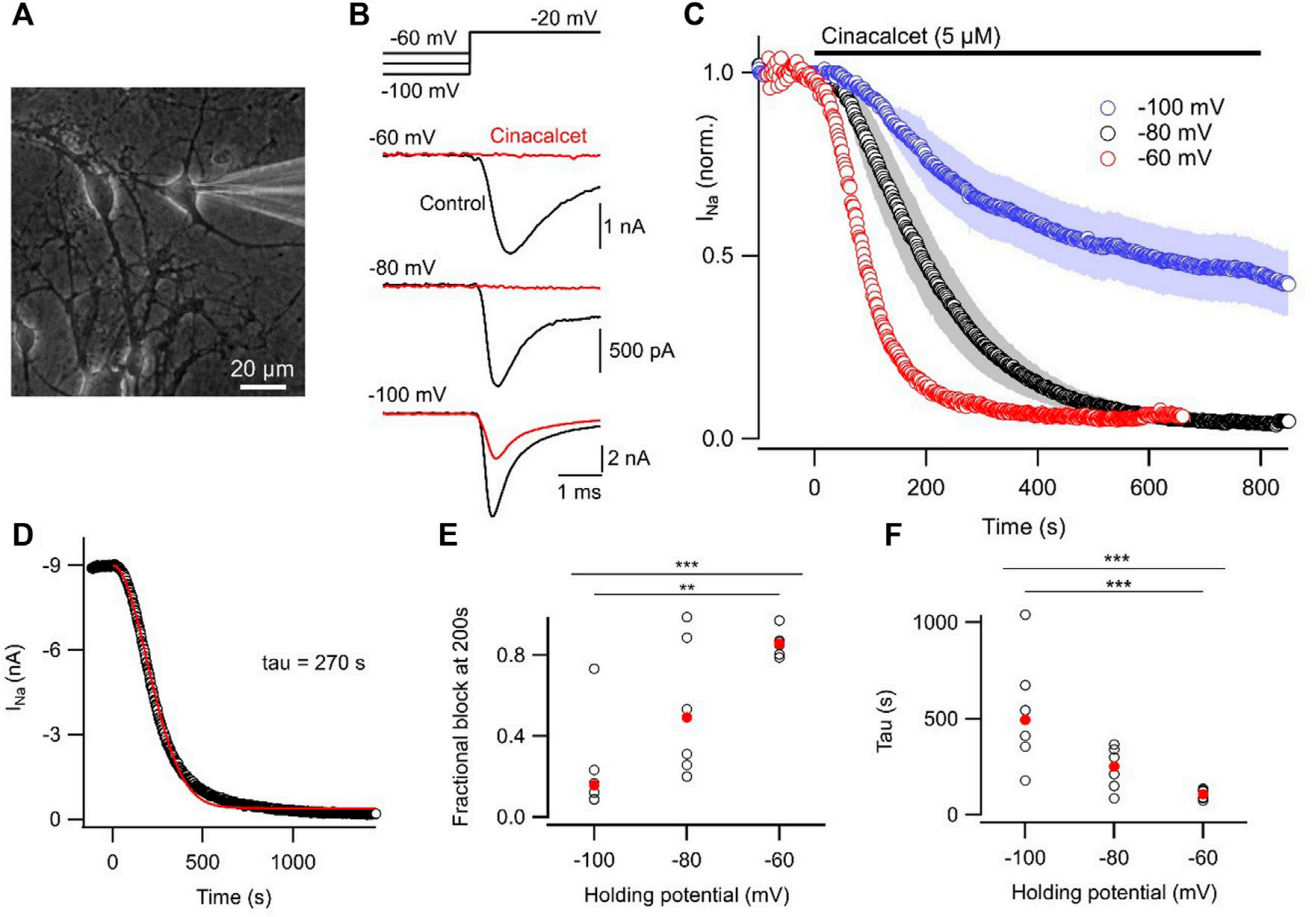
Inhibition of VGSC currents by cinacalcet is voltage-dependent. **(A)** Photomicrograph of neocortical culture with patch electrode in place. White bar indicates 20 µm. **(B)** Representative voltage traces show VGSC current baseline prior to (control, black) and following maximal inhibition by 5 µM cinacalcet (red). Cells were held at −60, −80, or −100 mV and a test pulse to −20 mV was elicited every 2 s. **(C)** Diary plot of average normalized peak VGSC current elicited by 20 ms test pulse every 2 s from a holding potential of -60 (red, n = 6), -80 (black, n = 6), or −100 mV (blue, n = 6). Control current baseline was established prior to cinacalcet addition at time 0. **(D)** Exemplar diary plot of peak VGSC current elicited as in **(B)** following application of 5 µM cinacalcet. Data fit with Eq. 1 shown in red. **(E)** Plot of individual (black, open circles) and median (red, filled circles) values of fractional inhibition at 200 s after application of 5 µM cinacalcet. Kruskal–Wallis test returned *p* < 0.001 overall, with *p* = 0.690, 0.003, and 0.090 for −60 mV versus −80 mV, −60 mV vs. −100 mV, and −80 mV vs. −100 mV respectively by Dunn’s corrected multiple comparisons. Here and in later Figures *p* values designated as follows: *p < 0.05, **p < 0.01, and ***p < 0.001. **(F)** Plot of individual (black, open circles) and mean (red, filled circles) values of tau (left). KW test returned *p* < 0.001 overall, with *p* = 0.189, 0.0008, and 0.192 for −60 mV versus −80 mV, −60 mV vs. −100 mV, and −80 mV vs. −100 mV respectively by Dunn’s corrected multiple comparisons.

Most pharmacological modulators of VGSC currents act directly on the ion channel and inhibit the sodium currents responsible for action potential generation. Like many VGSC inhibitors, cinacalcet appears to affect VGSC inactivation but is unusual because it acts indirectly, *via* a pathway mediated by G-proteins, to inhibit VGSC currents (Mattheisen et al., 2018). In total, we have observed substantial inhibition of VGSC currents in all of the >400 neocortical neurons we have tested. The neocortical cultures contain structurally diverse neurons and support cells (Figure 1A) which express a wide range of VGSC α and β subunits (Table 1). This indicates several VGSC isoforms are sensitive to cinacalcet-mediated inhibition. To more closely test if the pathway utilized by cinacalcet preferentially involves specific VGSC states, we compared the effect of holding potential, on the rate of cinacalcet-induced inhibition of VGSCs. In voltage clamp recordings, VGSC currents were elicited by test pulses to −20 mV (at 0.5 Hz) in cultured neocortical neurons perfused with Tyrode solution (containing 10 µM CNQX, 50 µM APV, and 10 µM Gabazine to block glutamatergic and GABAergic activity). After establishing a stable VGSC current baseline, 5 µM cinacalcet was applied to the neuron which reduced VGSC current amplitude (Figure 1). The time course of inhibition of VGSC currents by cinacalcet was described by a squared exponential function:

$$I\left(t\right)=Ae^{-{\left(t/\tau \right)}^{2}}+B$$ (1)

where I (t), A, B, t, and τ represent VGSC current amplitude during application, initial VGSC current amplitude, final VGSC current amplitude, time, and time constant respectively (Figure 1D). In contrast, directly-acting inhibitors produce a single exponential pattern of inhibition (Bean et al., 1983; Jo and Bean, 2017). The rate of inhibition was higher at depolarized holding potentials as illustrated by the average diary plots and confirmed both by the voltage-dependence of fractional inhibition at 200 s and the time constant of inhibition (Figures 1C–F). Hyperpolarization of the holding potential over the range of -60 to -100 mV decreased fractional block and increased τ substantially (Kruskal–Wallis test, both *p* < 0.001; Figures 1E, F). VGSC inhibition by cinacalcet was incomplete at −100 mV. The accelerated inhibition of sodium currents by cinacalcet at depolarized holding potentials probably reflects an increased affinity of the unidentified downstream inhibitory molecule to inactivated states of the VGSCs. These data support the proposal that inhibition of VGSC current by cinacalcet is voltage-dependent and occurs *via* an indirect pathway.

**TABLE 1:** Relative expression levels of VGSC subunit isoforms in neocortical cultures.

Subunit type	Gene symbol	Expression level (Log2)	Fraction of each isoform per subunit type (%)
α	Scn1a	3.72	3.0
α	Scn2a	7.54	42.4
α	Scn3a	6.31	18.1
α	Scn4a	4.88	6.7
α	Scn5a	4.77	6.2
α	Scn7a	4.8	6.4
α	Scn8a	5.72	12.0
α	Scn9a	3.39	2.4
α	Scn10a	2.73	1.5
α	Scn11a	2.46	1.3
β	Scn1b	11.34	39.5
β	Scn2b	6.51	1.4
β	Scn3b	11.91	58.7
β	Scn4b	4.7	0.4

### Cinacalcet shifts the voltage-dependence of fast inactivation

**FIGURE 2:**
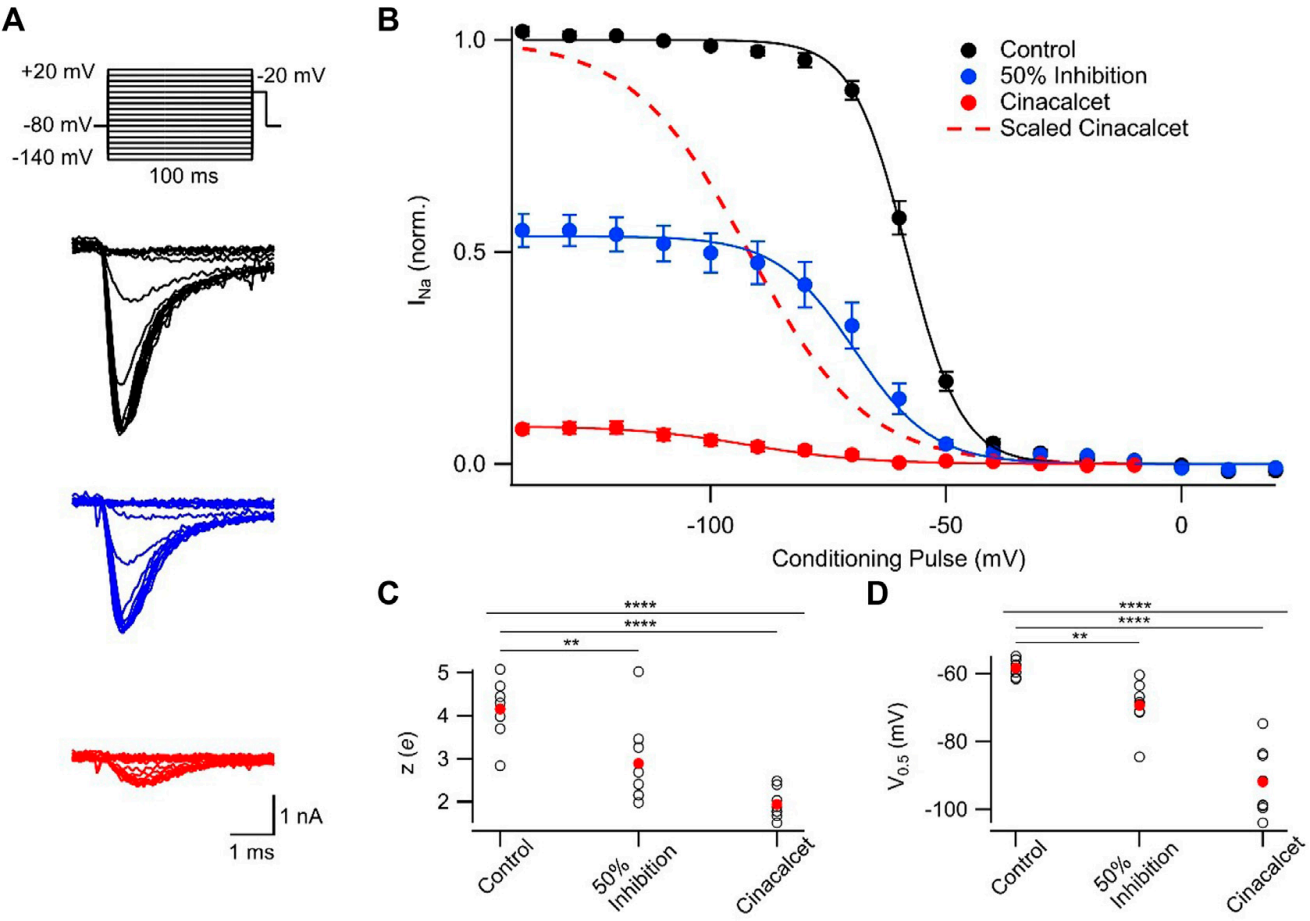
Cinacalcet shifted the voltage-dependence of fast inactivation. **(A)** Representative traces from a protocol used to isolate and evaluate the voltage-dependence of fast inactivation. Measurements were taken by applying a 100 ms depolarization to varying voltages (−140 to +20 mV in 10 mV increments) from a holding potential of −80 mV, and delivering a test pulse to −20 mV in control conditions (black), following inhibition of half of the starting current by 5 µM cinacalcet (blue), and following full inhibition (red). **(B)** Plot of average normalized peak VGSC currents elicited by test pulse following 100 ms conditioning pulse in control (black; n = 8), following inhibition of half of the starting current by cinacalcet (blue; n = 8), and following full inhibition (red; n = 8) fit to the Boltzmann equation. The 50% inhibition data were collected once cinacalcet had decreased the VGSC current elicited by the step from −80 mV to −20 mV, to 50% of control. Dashed red line indicates average cinacalcet fit normalized to the average control maximum. **(C)** Plot of individual (black, open circles) and mean (red, filled circles) apparent valence values. 1-W ANOVA RM test returned *p* < 0.0001 overall, with *p* = 0.0078 and < 0.0001 for control vs. 50% or control vs. full block respectively by Holm-Sidak corrected multiple comparisons. **(D)** Plot of individual (black, open circles) and mean (red, filled circles) V_0.5_ values. 1-W ANOVA RM test returned *p* < 0.0001 overall, with *p* = 0.003 and < 0.0001 for control vs. 50% or control vs. full block respectively by Holm-Sidak corrected multiple comparisons.

Preferential binding to a specific channel state will shift the dynamic equilibrium altering the relative fraction of VGSCs that occupy each state, as explained by the modulated receptor hypothesis (Hille, 1977; Hille, 1978; Bean et al., 1983). Initially we tested if VGSC recovery from fast inactivation was affected by cinacalcet. The currents were elicited by a test pulse to −20 mV following a family of 100 ms prepulses (−140 to 20 mV in 10 mV increments) and these control currents were compared with those activated following ∼50% or full inhibition by cinacalcet (5 μM; Figure 2A). Cinacalcet shifted the voltage-dependence of fast inactivation in the hyperpolarizing direction, with a mid-point (V_0.5_) of −58.3 ± 0.8 mV in control (n = 8), −69.4 ± 2.5 mV after ∼50% inhibition (n = 8), and −91.9 ± 3.6 mV following full inhibition (one-way ANOVA repeated measures, n = 8, *p* < 0.0001; Figures 2B, C). The hyperpolarizing prepulses facilitated recovery of only ∼10% of the fully inhibited VGSC currents from inhibition by cinacalcet (Figures 2A, B). Cinacalcet also decreased the apparent valence of the inactivation curves from 4.2 ± 0.2 *e* (control; n = 8), to 2.9 ± 0.4 *e* following ∼50% inhibition (n = 8), and 1.9 ± 0.1 *e* following full inhibition (1-W ANOVA RM, n = 8, *p* < 0.0001; Figure 2D). These data indicate that cinacalcet stabilized the fast-inactivated state of VGSCs.

### Cinacalcet stabilizes slowly recovering channel states

**FIGURE 3:**
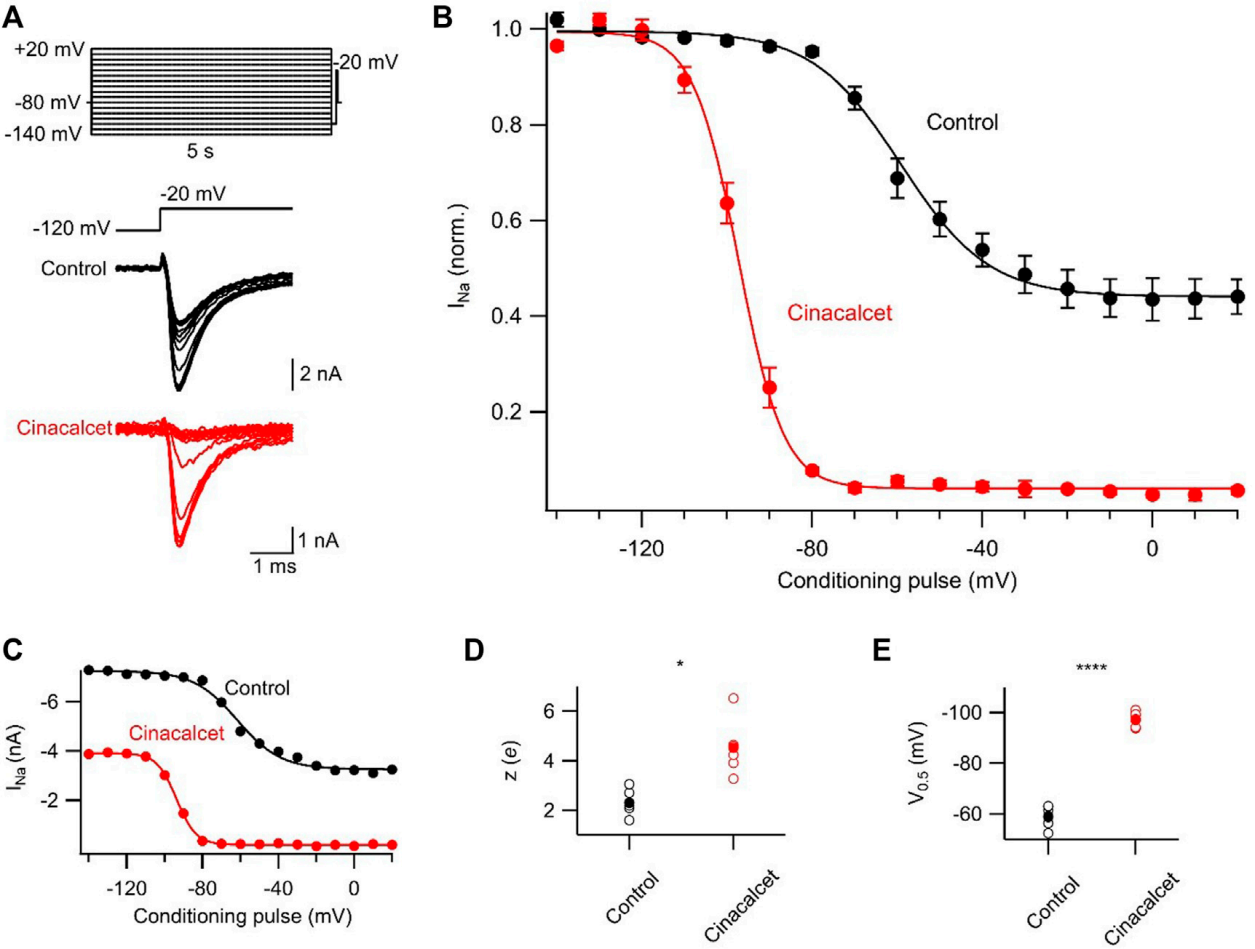
Cinacalcet enhancement of slowly recovering VGSCs. **(A)** Representative traces from a protocol used to isolate and evaluate the voltage-dependence of slow inactivation. Measurements were taken by applying a 5 s depolarization to varying voltages (−140 to +20 mV in 10 mV increments) from a holding potential of −80 mV, returning to −120 mV for 100 ms to allow recovery from fast inactivation, and delivering a test pulse to −20 mV in control conditions (black) and following full inhibition by 5 µM cinacalcet (red). **(B)** Plot of average normalized peak VGSC currents elicited by test pulse following 5 s conditioning pulse and 100 ms recovery period in control (black; n = 6) and following full inhibition by cinacalcet (red; n = 6) fit to the Boltzmann equation. Dashed red line indicates average cinacalcet fit normalized to the average control maximum. **(C)** Plot of individual (black, open circles) and mean (red, filled circles) V_0.5_ values. **(D)** Plot of individual (black, open circles) and mean (red, filled circles) apparent valence values.

To further investigate the inhibitory mechanism of cinacalcet, we explored the possibility that X interacts with the slow-inactivated state of the VGSC. A test pulse to −20 mV, delivered after a 100 ms interval at −120 mV, evoked VGSC currents following a series of 5 s prepulses (−140 to +20 mV in 10 mV increments; Figure 3A). The 100 ms interval at −120 mV facilitated recovery from fast inactivation, so that the test pulse permitted the comparison of the effects of the prepulse on VGSC current recovery from a slow-inactivated state. Slow inactivation was complete at −10 mV in control with ∼45% of VGSC currents still available for activation (Figure 3). In the presence of 5 µM cinacalcet, we observed a substantial increase in the fraction of channels recovering slowly (96 ± 0.6%; n = 6) compared to control (56 ± 4%; n = 6; Figure 3B). We also observed a significant increase in the rate of slow inactivation development, with theapparent valences increasing from 2.3 ± 0.2 *e* in control (n = 6) to 4.5 ± 0.5 *e* following inhibition by cinacalcet (n = 6, *p* = 0.004; Figure 3C). In addition, the voltage-dependence of slow inactivation shifted in the hyperpolarizing direction in the presence of cinacalcet, with a midpoint of −59.2 ± 1.7 mV in control and −97.3 ± 1.2 mV (*p* = 0.004) following inhibition by cinacalcet (Figure 3D). Taken together, these results indicate that cinacalcet apparently stabilizes the slow-inactivated VGSC state.

### Cinacalcet-mediated inhibition occurs more rapidly than slow inactivation of VGSC currents

**FIGURE 4:**
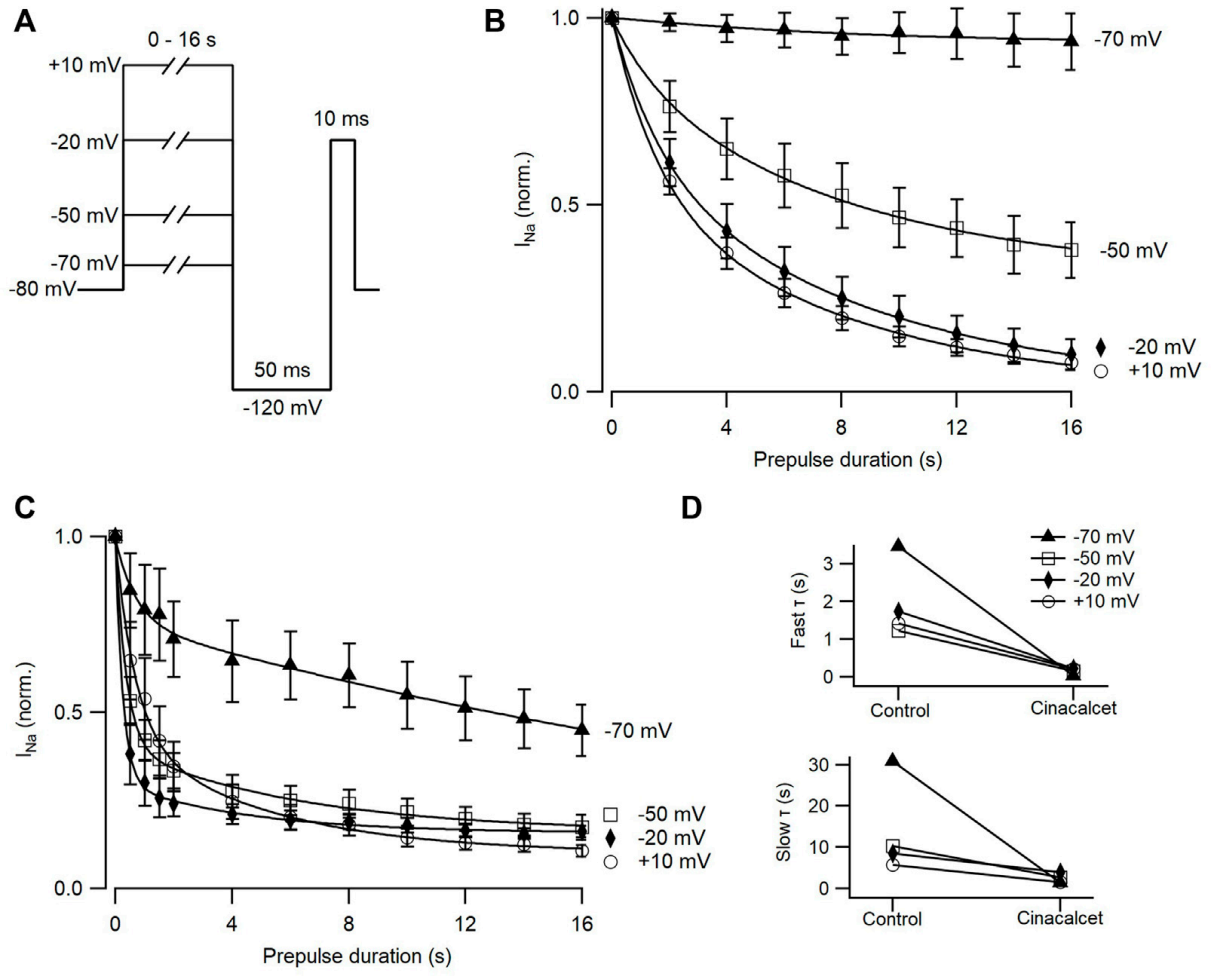
Cinacalcet affinity does not correlate with development of slow inactivation. **(A)** Voltage protocol used to show development of slow inactivation with increased time at various voltages. Measurements were taken by applying a variable time step to either −70, −50, −20, or +10 mV from a holding potential of −80 mV, and delivering a test pulse to −20 mV following a 50 ms recovery period at -120 mV. **(B)** Time course of development of slow inactivation in control conditions, with conditioning pulses as in A from −70 (filled triangle; n = 5), -50 (open square; n = 5), −20 (filled diamond; n = 5), or +10 mV (open circle; n = 5) fit to a double exponential. Currents were normalized to the first current at each voltage when there was no inactivating pulse. **(C)** Time course of development of slow inactivation following complete inhibition by 5 µM cinacalcet, with conditioning pulses as in A from −70 (filled triangle; n = 6), −50 (open square; n = 6), −20 (filled diamond; n = 6), or +10 mV (open circle; n = 5) fit to a double exponential. Currents were normalized to the first current at each voltage when there was no inactivating pulse. **(D)** Plot of average fast (top) and slow (bottom) τ values in control and following inhibition by cinacalcet.

We attempted to further characterize the kinetics of inhibition by cinacalcet in our neocortical neurons using voltage protocols designed to differentiate between the relative affinities for fast and slow-inactivated VGSC states (Kuo and Bean, 1994; Errington et al., 2008). The presence of channels in the slow-inactivated state was measured with a variable length (0–16 s) prepulse to either −70, −50, −20, or +10 mV, followed by a brief recovery period at −120 mV to allow recovery from fast inactivation, and a test pulse to -20 mV (Figure 4A). Figure 4B shows the induction of slow inactivation at different voltages in the absence of cinacalcet. There was minimal slow inactivation at −70 mV; almost all VGSCs recovered from inactivation during the 50 ms recovery period even after 16 s at −70 mV. The occupancy of the slow-inactivated state increased as the inactivating pulse was made more positive, reaching ∼93% with a 16 s pulse at +10 mV.

Figure 4C shows the development of VGSC current inhibition during application of 5 µM cinacalcet at different depolarized voltages. The inhibition by cinacalcet can be compared directly with the rate and voltage dependence of slow inactivation shown in Figure 4B, because the pulse protocols are identical and the individual experiments were paired. Importantly, however, the 50 ms pulse to −120 mV that eliminates fast inactivation in the control setting may not have the same effect during inhibition by cinacalcet. If fast-inactivated VGSCs become bound during the variable length prepulse, then the 50 ms pulse to −120 mV will hypothetically only recover those fast-inactivated channels that are *unbound*. Thus, in control, the test pulse assays channels that are in the slow-inactivated state whereas in the presence of cinacalcet, the test pulse will also assay channels that are bound by X.

At each voltage, the kinetics of inhibition by cinacalcet were substantially faster than the development of slow inactivation. At −70 mV, cinacalcet inhibition developed with a fast time constant of 0.03 s and a slow time constant of 1.46 s whereas slow inactivation developed with a fast time constant of 3.46 s and a slow time constant of 30.9 s (Figure 4D). At −50, −20, and +10 mV, cinacalcet inhibition developed with fast time constants of 0.16, 0.23, and 0.20 s respectively, and slow time constants of 2.7, 3.9, and 1.5 s respectively. Conversely, slow inactivation developed with fast time constants of 1.2, 1.7, and 1.4 s at −50, −20, and +10 mV, respectively, and slow time constants of 10.3, 8.5, and 5.7 s. The results with a prepulse to −70 mV demonstrate most clearly the lack of selective binding of X to the slow-inactivated state. There was minimal slow inactivation even with a prepulse to −70 mV for 16 s, yet there was substantial inhibition by cinacalcet (∼55%). These results indicate that cinacalcet does not promote the binding of X exclusively to the slow-inactivated state, as development of inhibition could never be faster than the development of slow inactivation if this were the case.

### Inhibition of VGSC currents by cinacalcet is accelerated at voltages favoring the fast-inactivated state

**FIGURE 5:**
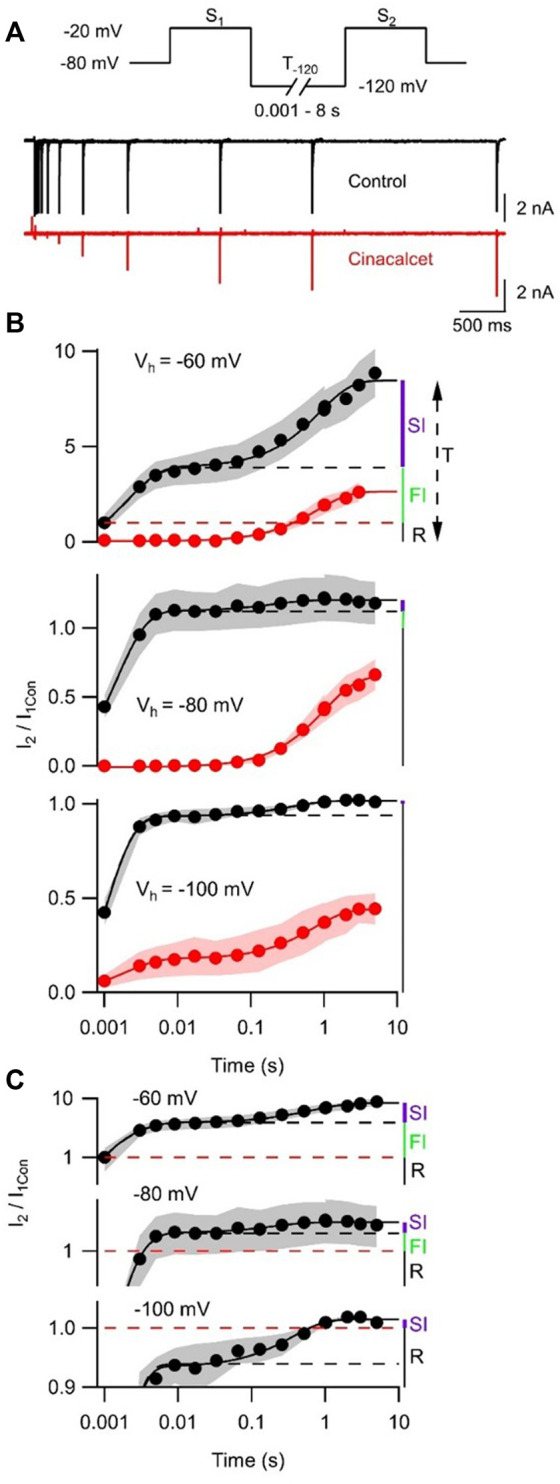
Cinacalcet inhibition is reversible by prolonged hyperpolarization. **(A)** Superimposed representative traces from a double pulse protocol (S1 and S2) used to elicit VGSC currents in control (top, black) or after complete inhibition by 5 µM cinacalcet (bottom, red) in the whole cell from a holding potential (V_h_) of −80 mV. Test pulses S1 and S2 are 10 ms in length and separated by a variable-length recovery period at −120 mV. **(B)** Graph showing double-exponential increase in VGSC current amplitude with increased time at −120 mV in control conditions (black) (Τ1 = 2.1 ± 0.5 ms, Τ2 = 0.87 ± 0.12 s; n = 4) and single-exponential recovery of VGSC current after full inhibition with 5 µM cinacalcet (red) with increased time at -120 mV (Τ = 0.82 ± 0.05 s; n = 4); from a holding potential of -60 mV (top panel) in the whole cell. Graph showing double-exponential increase in VGSC current amplitude with increased time at −120 mV in control conditions (black) (Τ1 = 1.4 ± 0.1 ms, T2 = 206 ± 62 ms; n = 4) and single-exponential recovery of VGSC current after full inhibition with 5 µM cinacalcet (red) with increased time at −120 mV (Τ = 1.02 ± 0.05 s; n = 5); from a holding potential of −80 mV in the whole cell (middle panel). Graph showing double-exponential increase in VGSC current amplitude with increased time at −120 mV in control conditions (black) (Τ1 = 0.92 ± 0.06 ms, T2 = 0.37 ± 0.07 s; n = 4) and double-exponential recovery of VGSC current after full inhibition with 5 µM cinacalcet (red) with increased time at −120 mV (Τ1 = 1.93 ± 0.31 ms, Τ2 = 0.73 ± 0.05 s; n = 4); from a holding potential of −100 mV in the whole cell (bottom panel). Currents are normalized to current elicited by step from each respective holding potential to −20 mV before cinacalcet addition and to equivalent IV step, in cinacalcet and control traces, respectively. **(C)** Graph showing control data from **(B)** with logarithmic transformation and expansion of y-axis to emphasize the relative sizes of SI, FI, and R states at the three holding potentials.

Previous work has shown that sustained hyperpolarization slowly reverses cinacalcet-mediated inhibition of VGSC currents (Mattheisen et al., 2018). We tested if holding potential (−60, −80, or −100 mV) strongly affected the dynamics of recovery from inhibition. A double-pulse protocol (S_1_ and S_2_, each to −20 mV for 10 ms) was used to elicit VGSC currents (I_1_ and I_2_) in control or after complete inhibition by 5 µM cinacalcet (Figure 5A). The ratio I_2_/I_1Con_ reflected the amplitude of the VGSC current elicited by S_2_ compared to that elicited by the step to −20 mV from the holding potential before drug application. At −80 mV, I_2_/I_1Con_ recovered fully within 10 ms before cinacalcet application (Figure 5B). Cinacalcet attenuated and slowed the recovery of I_2_/I_1Con_ substantially, so that it eventually reached between 31% and 53% of the control I_2_/I_1Con_ after 8 s at −120 mV (Figure 5B). The majority of this recovery was well described by a single exponential where τ was between 1 and 1.9 s (red curves, Figure 5B). The holding potential had a much greater effect on the dynamics of the gating of the control VGSC currents than the recovery following inhibition by cinacalcet.

Models that describe complex VGSC function indicate the channels can occupy multiple fast and slow inactivation states (Ulbricht, 2005; Milescu et al., 2010; Goldfarb, 2012; Cervenka et al., 2018). The indirect mechanism of inhibition by cinacalcet adds further complexity. To reduce the number of parameters we described the action of cinacalcet using a simpler model with only single fast and slow inactivation states. Two exponential phases of recovery of I_2_/I_1Con_ that represent VGSC repriming (Figures 5B,C, black circles), were observed in the absence of cinacalcet and represent the fast and slow inactive states. The amplitudes of the total I_2_/I_1Con_ value (T, upper panel Figure 5B) and the amplitudes of each exponential were used to estimate the fraction of VGSCs in resting (R, black), fast-inactivated (FI, green), and slow-inactivated (SI, purple) states. The holding potential substantially changed the fraction of VGSCs in the resting state, which corresponded to a I_2_/I_1Con_ value of unity (red broken line, Figure 5B), so that R decreased relative to T as holding potential was depolarized - and, the fraction of VGSCs in the resting state was 1/T. The FI component was defined as any component of I_2_/I_1Con_ below the asymptote for the faster exponential fit (black broken) above the resting component (I_2_/I_1Con_ = 1, broken red line). The SI component was the difference between the asymptote to the double exponential fit (black solid) and the higher of the asymptote of the faster exponential fit (black broken) or I_2_/I_1Con_ = 1. The fraction (*F*) of the VGSCs in each state was obtained by dividing each component by T (*F*_*R*_ *, F*_*FI*_ *, and F*_*SI*_) at each value of V_h_. Using the law of mass action and the observation that reversal of VGSC inhibition was relatively slow [Figure 5B and (Mattheisen et al., 2018)], the rate of inhibition of VGSCs (dB/dt) by the unidentified inhibitory molecule was directly proportional to the sum of the products of *F* and *k* for each channel state:

$$\frac{dB}{dt}\;\alpha \;\left[X\right]\;\left(\;F_{R}.\;k_{R}+F_{FI}.\;k_{FI}+F_{SI}.\;k_{SI}\;\right)$$

where [X], represents the unknown concentration of inhibitory molecule, and k represents the association constants for the three VGSC states. By incorporating a constant, C:

$$\frac{dB}{dt}=\left[X\right]\;\left(F_{R}.\;k_{R}+F_{FI}.\;k_{FI}+F_{SI}.\;k_{SI}\;\right)\;C$$ (2)

Using Eq. 2, the voltage-dependent rates of inhibition (1/ τ , Figure 1F), and the values of F for each of the states at V_h_ (Table 2) we constructed a simultaneous equation for each of the three holding potentials. These three equations were solved to return the relative association rates for the R, FI, and SI states of 1, 10.1, and 2.3 respectively.

**TABLE 2:** Parameters used in Model.

V_h_ (mV)	R	FI	SI	1/ τ (s^−1^)
−60	0.118	0.342	0.540	0.0096
−80	0.832	0.100	0.069	0.0040
−100	0.985	0	0.015	0.0020

### Cinacalcet state-dependent inhibition of VGSC currents confers non-linear spike block

**FIGURE 6:**
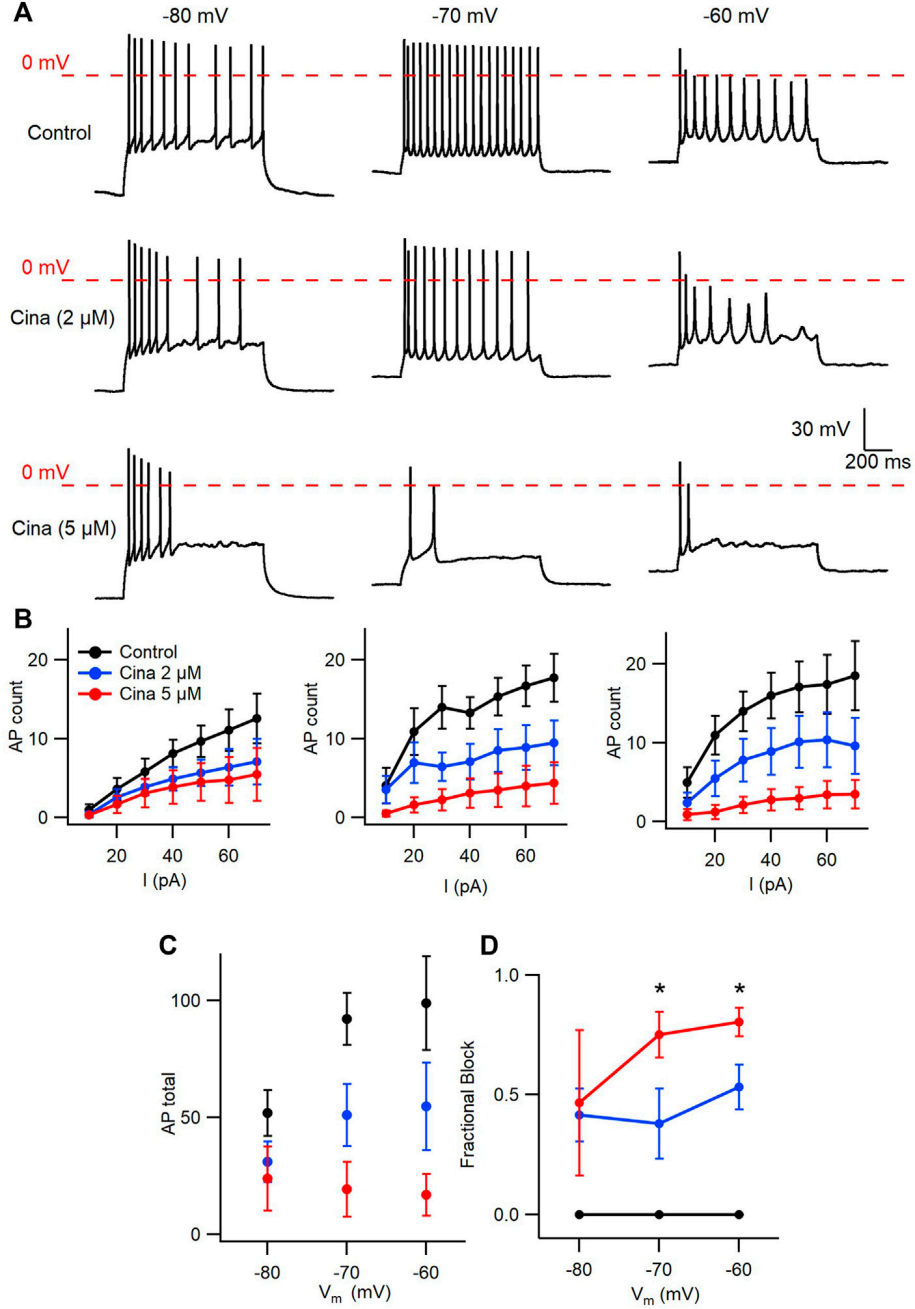
Voltage-dependent inhibition of spike generation by cinacalcet. **(A)** Exemplar traces showing voltage- and concentration-dependent inhibition by cinacalcet. Three whole-cell current clamp recordings with holding potentials at −80 mV, −70 mV and −60 mV. Measurements were taken at 70 pA before and after cinacalcet was applied for 5 min at incremental doses of 2 μM and 5 µM. **(B)** Action potential (AP) count following serial current injections from 10 pA to 70 pA for 1 s at holding potentials of −80 mV, −70 mV and −60 mV (from left to right) showing decrease in average AP number with increasing cinacalcet dose from control (black), 2 µM (blue) and 5 µM (red) as well as increased separation of the curves as more depolarized holding potentials. **(C)** Plot demonstrating a comparison of the cumulative number of APs as seen in **(B) (D)** Fractional Inhibition of AP during maximal current injection of 70 pA at each holding potential. The fractional inhibition with 5 µM cinacalcet increased from 46% to 75%–80% with depolarization of holding potential from −80 mV to −70 mV to −60 mV. In addition the dose-dependence of the fractional inhibition increased in a non-linear manner with no difference at −80 mV but showed a significant increase in inhibition at −70 mV and −60 mV with increasing cinacalcet dose (mean fractional inhibition 42 ± 11% vs. 47 ± 30% at −80 mV, 38 ± 15% vs. 75 ± 10% at −70 mV, and 53 ± 9% vs. 80 ± 6% at −60 mV at 2 µM and 5 μM; n = 10–11 for all groups; **p <0.01 by paired t-test).

Cinacalcet binding to an unidentified receptor triggers a pathway resulting in the generation of an inhibitory molecule that preferentially inhibits the fast-inactivated VGSC state. Action potentials are near digital signals in contrast to the graded VGSC current amplitude. The non-linearity of action potentials and the complexity of action of cinacalcet, make it hard to predict how the pathway modulated by cinacalcet will impact action potential generation. We tested how cinacalcet impacted excitability in neocortical neurons at membrane potentials between -60 and -80 mV. Whole-cell current clamp recordings were performed in the presence of (in µM) 10 CNQX, 10 Gabazine, and 50 APV to prevent confounding by the actions of cinacalcet on synaptic transmission. Resting membrane potentials of neurons in this recording configuration were −74.3 ± 1.1 mV (n = 30). Action potentials were elicited by a series of one second current injections (10–70 pA) in neurons current-clamped close to -80 mV (Figure 6A, left panel). Thereafter the recording mode was switched to voltage clamp, to facilitate a consistent holding potential, and 2 µM cinacalcet applied at −80 mV. After 5 min, the recording mode was switched back to current clamp and depolarizing pulses injected from a target holding potential of −80 mV. Cinacalcet was increased to 5 µM and the process repeated as before (Figure 6A, left column). Action potential number decreased at each current injection as cinacalcet concentration was increased and these effects were larger in separate recordings where the membrane potential was targeted to -70 or −60 mV (Figures 6A, B). At each target holding potential, the measured values were well-matched between the control and cinacalcet groups (Table 3). A similar striking effect was observed by comparing the sums of action potentials generated at each holding potential (Figure 6C). Action potential number increased with depolarization of the holding potential and cinacalcet reduced the total number of action potentials generated by the series of current injections.

**TABLE 3:** Action potential characteristics.

Target holding potential (n)	[Cinacalcet] (µM)	Recorded hold. pot. (mV)	Input res. (MΩ)	Action pot. threshold (mV)	Action pot. amp. (mV)	Action pot. half-width (ms)
−80 mV (9)	0	−80.2 ± 1.2	482 ± 49	−47.6 ± 1.6	67.9 ± 1.8	1.4 ± 0.7
	2	−80.3 ± 0.8	493 ± 35	−49.4 ± 1.6	64.5 ± 2.0	1.4 ± 0.9
	5	−81.2 ± 0.5	506 ± 45	−48.5 ± 3.5	55.8 ± 5.1	1.7 ± 0.1
−70 mV (11)	0	−70.6 ± 0.6	347 ± 32	−45.1 ± 1.2	64.9 ± 1.7	1.5 ± 0.1
	2	−70.5 ± 0.3	354 ± 32	−45.4 ± 1.3	58.3 ± 4.0	1.8 ± 1.9
	5	−70.9 ± 0.3	349 ± 31	−44.2 ± 1.8	44.2 ± 4.3	2.5 ± 0.3
−60 mV (10)	0	−63.4 ± 1.3	265 ± 48	−44.5 ± 1.6	65.5 ± 3.9	2.1 ± 0.3
	2	−61.9 ± 0.7	244 ± 33	−44.2 ± 1.4	56.5 ± 4.9	3.2 ± 1.1
	5	−61.1 ± 0.7	243 ± 30	−40.8 ± 2.2	45.8 ± 5.5	4.0 ± 1.3
Test			2W- ANOVA RM	Mixed effects model	Mixed effects model	Mixed effects model
Probability of interaction			0.598	0.336	<0.001	0.236
Probability of cina. effect			0.969	0.191	<0.001	0.009
Probability of hold. pot. effect			<0.001	0.100	<0.001	0.130

Using the number of spikes elicited by the 70 pA current injection we examined how the voltage-dependence of cinacalcet inhibition of sodium conductances (Figure 1) impacted action potential generation (Figure 6D). While the average fractional block of action potentials was the same for 2 and 5 µM cinacalcet at −80 mV (42 ± 11% vs. 47 ± 30%), the relative block of the two concentrations increased substantially at −70 mV (38 ± 15% vs. 75 ± 10%). Further depolarization to -60 mV reduced the difference in fractional block between the two concentrations of cinacalcet (53 ± 9% vs. 80 ± 6%). Overall, stronger depolarizations increased the potency of action potential block by cinacalcet.

In addition to reducing the likelihood of action potential generation, VGSC inhibition by cinacalcet may reduce the rate of depolarization and height of the action potential resulting in a shorter and broader waveform. Depolarization may also impact spike waveform by causing VGSC inactivation and changing other voltage-gated channel availability. Input resistances and the properties of the action potentials (the first elicited by a 70 pA depolarizing current) are documented in Table 3 (Mean ± SEM). A two-way ANOVA with repeated measures (2-W ANOVA RM) was performed to analyze the effect of the target holding potential and cinacalcet treatment on input resistance (Table 3) and revealed no interaction (F (4, 54) = 0.696, *p* = 0.598). Main effects analysis showed holding potential (*p* < 0.001) but not cinacalcet treatment (*p* = 0.969) had a significant effect on input resistance. These results suggest cinacalcet did not substantially block ion channels already open at the holding potentials but that conductances were activated by depolarization of the holding potential.

In some recordings, action potentials were not generated following treatment with cinacalcet or depolarization resulting in “missing values”. Consequently, we used mixed-effects analyses to determine the effect of the target holding potential and cinacalcet treatment on the action potential properties. As shown, (Table 3) action potential amplitude and half-width were both affected. Cinacalcet treatment and holding potential interacted to affect action potential amplitude (F (4, 48) = 180.1, *p* <0.001). Main effects analyses showed that both factors independently affected action potential amplitude (both *p* < 0.001). There was not a significant interaction of cinacalcet treatment and holding potential on action potential half-width (F (4, 48) = 1.439, *p* = 0.236). Cinacalcet treatment, but not holding potential, had a significant effect on half-width (*p* = 0.009 and *p* = 0.130). The target holding potential and cinacalcet treatment did not interact to affect action potential threshold (F (4, 48) = 1.169, *p* = 0.336) or affect threshold independently (*p* = 0.100 and = 0.191, respectively). The smaller, broader action potentials observed at lower frequency following cinacalcet treatment are consistent with VGSC inhibition.

## Discussion

Cinacalcet inhibits VGSC currents strongly in the vast majority of neocortical and hippocampal neurons (Mattheisen et al., 2018). Characterizating the mechanism of this prevalent and high-efficacy inhibition will help determine its role in regulating cortical excitability. Here we demonstrate how cinacalcet inhibits the VGSC current by activating a downstream inhibitory molecule that preferentially binds to the fast-inactivated state, how this stabilizes the inactivated states, and how this impacts neuronal excitability in a non-linear manner. Our findings that all neurons tested in our mixed neocortical cultures, responded to cinacalcet (n > 400) and that the cultures expressed a broad range of VGSC isoforms (Table 1) indicate the signaling pathway is positioned to modulate many VGSC subtypes.

In our investigation of inactivated VGSC state preference, we used voltage protocols designed to evoke and study the fast- and slow-inactivated states, and the ways in which these states are shifted by the addition of cinacalcet (Figure 2 and Figure 3). Each inactivation curve was shifted in the hyperpolarizing direction by the addition of cinacalcet, indicating stabilization of the inactivated state, and the addition of cinacalcet greatly enhanced the proportion of channels recovering slowly (Figure 3). We found that fast and slow inactivation were both shifted significantly after inhibition with 5 µM cinacalcet by −33 and −38 mV respectively (Figure 2 and Figure 3). The voltage dependence of VGSC current inhibition by cinacalcet is characteristic of many sodium channel inhibitors, and can be understood by the modulated receptor model (Hille, 2001) which describes how preferential binding to a specific channel state disturbs the dynamic equilibrium, causing a counteracting shift and new position of equilibrium. The principle of microscopic reversibility ensures that tighter binding to the inactivated state by the inhibitory molecule results in a greater fraction of the uninhibited channels residing in the inactive state at that voltage, corresponding to a hyperpolarizing shift in V_0.5_ (Hille, 1977; Hille, 1978; Bean et al., 1983). To further distinguish between the possibilities of selective binding to the slow-inactivated state and slow binding to the fast-inactivated state, we used a protocol to investigate the kinetics of slow inactivation as previously described (Kuo and Bean, 1994) (Figure 4). The results of this experiment argue against the possibility of selective binding to the slow-inactivated state, as the development of inhibition by cinacalcet proceeds at a rate faster than the development of slow inactivation. However, interpretation of data obtained with this approach may not be so straightforward. For example, with this approach it is difficult to distinguish VGSC recovery from inhibition if the channels are in the slow-inactivated or fast-inactivated states when the dissociation of the inhibitory molecule is relatively slow (Karoly et al., 2010). Since cinacalcet acts indirectly to inhibit VGSCs it seems unlikely that external concentrations of cinacalcet are linearly related to the concentration of the downstream inhibitory molecule. In the absence of the information about the effects of changing the concentration of the inhibitory molecule the estimation of the relative affinity of the inhibitory molecule for the R, FI, and SI states using Eq. 2, required a number of simplifying assumptions. The normalized inward currents elicited by the double pulse protocol before and after perfusion of cinacalcet were plotted at three separate holding potentials (Figure 5). We used a rate constant derived from the median rates of inhibition at these holding potentials and utilized simplifying assumptions such as relatively slow off rate for the inhibitory molecule, a relatively abrupt increase and stable concentration of inhibitory molecule following the application of cinacalcet, that all VGSC isotypes respond similarly to cinacalcet application, and that interconversions between channel states are relatively rapid compared to the actions of the inhibitory molecule. Another assumption incorporated is that the multiple VGSC isoforms expressed in the neocortical neurons behave similarly following cinacalcet inhibition. Using Eq. 2 and these assumptions, we estimated the relative affinities for the various states were FI:SI:R in the ratio 10.1 : 2.3: 1. The accuracy of these predictions will be tested as other components in this pathway are identified thereby allowing the development and use of more conventional multi-state models (Karoly et al., 2010). The complexity provided by the voltage-dependence of VGSC inhibition by cinacalcet, manifests as reduced excitability overall and broadening and shortening of residual action potentials (Figure 6; Table 3). All of these changes are sensitive to the membrane potential and so will lead to use-dependence or increased apparent efficacy during times of neuronal activity.

We have proposed a mechanism of action whereby cinacalcet binds to an unidentified receptor triggering a pathway resulting in the generation of an inhibitory molecule that preferentially inhibits the fast-inactivated VGSC state. It has been pointed out, that the voltage-dependence we observed could arise from another source upstream of the VGSC if that process is voltage-dependent. Using the modulated receptor hypothesis, the shifts in VGSC gating characteristics we observed following inhibition (Figure 2, Figure 3 and Figure 5), indicate that the inhibitory molecule is not binding equally to the various VGSC states (Hille, 1977; Hille, 1978; Bean et al., 1983). However, we cannot rule out the possibility that some of the voltage-dependence we observed arises upstream of the VGSC.

As mentioned above, CaSR is not the GPCR transducing the action of cinacalcet and the identity of the cinacalcet target remains unclear. CaSR interacts with the GABA_**B**_ receptor in some cells (Chang et al., 2020) but this receptor did not contribute to inhibition of VGSC currents by cinacalcet (Mattheisen et al., 2018). The muscarinic acetylcholine receptor M1, dopamine receptor D1, and metabotropic receptor mGluR1 have all been identified as GPCRs that can regulate VGSC currents in the cortex *via* PKA or PKC (Cantrell et al., 1996; Cantrell et al., 1997; Carlier et al., 2006) but agonists and antagonists operating *via* these receptors did not modulate VGSC currents that were sensitive to cinacalcet (Mattheisen et al., 2018). Nor did a wide range of blockers of PKA and PKC, indicating that cinacalcet is operating *via* a different mechanism than those utilized by acetylcholine, dopamine, and glutamate. The pathway utilized by cinacalcet to modulate VGSC currents also has a higher efficacy and slower timecourse than those activated by acetylcholine, dopamine, and glutamate. For instance the rates of inhibition and reversal of inhibition by cinacalcet are more than an order of magnitude slower than muscarinic agonists (Figure 1) (Cantrell et al., 1996; Mattheisen et al., 2018). Consequently, activation of the pathway used by cinacalcet will provide a much slower pattern of modulation of neuronal excitability. The shift in gating of slow and fast inactivation by cinacalcet was more than -30 mV (Figures 2, 3) whereas gating was unaffected by dopamine agonists (Cantrell et al., 1997). This higher voltage-dependence of inhibition by cinacalcet will result in cinacalcet impacting excitable cells that are depolarized much more than those that are hyperpolarized. Comparable differences in the voltage dependence of ion channel inhibitors has been shown to result in enormous differences in tissue-specific potency. A vivid example is provided by dihydropyridines where at therapeutic levels the L-type cardiac calcium channels are unaffected whereas those in relatively depolarized smooth muscle cells are blocked (Bean, 1984).

The high dynamic range and abundance, positions the pathway utilized by cinacalcet to inhibit VGSC currents, to be able to contribute strongly to neuronal plasticity. However, it remains unclear under what physiological conditions the signaling pathway impacts neuronal excitability. Since cinacalcet stabilizes the VGSC inactive state(s), after which the VGSCs only move to the resting state after a prolonged, strong hyperpolarization (Figure 5), the holding potential-dependent fraction of the VGSC current represented by slow inactivation presumably reflects the upper limit of activity of the pathway under basal conditions (Table 2). Once available, specific inhibitors that block the effects of cinacalcet on VGSCs could be used to address this question directly. Currently, the degree of basal activity is unclear. GDPβS did not prevent “run-down” of VGSC currents suggesting there was no basal stimulation of the pathway in the absence of cinacalcet (Mattheisen et al., 2018). In contrast, GDPβS did slightly depolarize the inactivation gating in the absence of cinacalcet, consistent with a modest level of basal activity.

As discussed previously, the doses of cinacalcet consumed by patients lead to serum levels of 50 nM which is only expected to inhibit 2% of the VGSC current based on its concentration-effect relationship (Mattheisen et al., 2018). However, cinacalcet has a volume of distribution of >1,000 L indicating it may be concentrated in the brain and reach levels >50 nM (Agency E.M., 2022). However, it is important to note that even if cinacalcet inhibits only 2% of the brain VGSC currents, this would be expected to have a clinical effect on excitability. In comparison, the antiepileptic drug phenytoin is therapeutic with a total serum level of 20 μg/ml (corresponding to a CSF phenytoin of 0.14 μg/ml or 0.6 µM) (Brodie et al., 1985; Kane et al., 2013), yet only blocks 1%–4% of VGSCs at this level. If cinacalcet accumulates in the brain even a little, we predict that the inhibition of VGSC currents will decrease the likelihood of action potential firing in many neuronal circuits and so lead to noticeable changes in behavioral and clinical effects comparable to high doses of phenytoin. Concurrent with changes arising from VGSC inhibition, stimulation of brain CaSR by cinacalcet will also increase and decrease spontaneous and evoked neurotransmission respectively (Phillips et al., 2008; Vyleta and Smith, 2011). These additional changes would be expected to unbalance levels of neuronal excitability, possibly impacting homeostatic plasticity that regulates activity within brain regions (Li et al., 2020). It has been proposed that CaSR at nerve terminals has a homeostatic role to minimize the impact of dynamic physiological or pathological changes in external calcium (Smith et al., 2004; Chen et al., 2010). Stimulation of nerve terminal CaSR by cinacalcet may impair the ability of the terminal to sustain release during times of activity. In patients with underlying hyperparathyroidism, the overall action of cinacalcet on brain function will be even more complex. The inhibition of VGSC currents by cinacalcet will be confounded by its beneficial effects on calcium and magnesium levels (Nemeth et al., 2004) which will increase neuronal excitability (Martiszus et al., 2021). By reducing external calcium levels in the brains of patients, cinacalcet is predicted to also modify calcium-dependent short term plasticity (Zucker and Regehr, 2002; Vyleta and Jonas, 2014). The complexity of these interacting cinacalcet-sensitive pathways increases the likelihood that the drug will modify overall behavior.

We have determined that the action of cinacalcet is highly state dependent and that VGSC inhibition is favored especially when the fast-inactivated state is more preponderant. This state dependence of cinacalcet’s effect, manifested as use-dependent inhibition (Mattheisen et al., 2018) and strong dependence of action potential block on the neuronal membrane potential. The prevalence and efficacy of the signaling pathway by which cinacalcet inhibits VGSC, positions it to inhibit neocortical neuronal excitability non-uniformly, with major impact on active circuits containing more depolarized neurons. Combined with the unusual pattern of use-dependence (Mattheisen et al., 2018) and the large difference in rate of inhibition over the resting membrane potential range (Figures 1, 6) it is likely that cinacalcet will alter cell excitability differently to many other sodium channel inhibitors. Further identification of the molecular components of the pathway will facilitate the development of analogous ligands that may avoid co-stimulation of the CaSR and be useful additions to the armamentarium of therapeutic sodium channel inhibitors.

## Data Availability

The datasets presented in this study can be found in online repositories. The names of the repository/repositories and accession number(s) can be found below: https://www.ncbi.nlm.nih.gov/bioproject/?term=GSE218028.
